# Lamb Age has Little Impact on Eating Quality

**DOI:** 10.3390/foods9020187

**Published:** 2020-02-13

**Authors:** Claire E. Payne, Liselotte Pannier, Fiona Anderson, David W. Pethick, Graham E. Gardner

**Affiliations:** 1 Engineering and Education, College of Science, Health, Murdoch University, Perth 6150, Australia; L.Pannier@murodch.edu.au (L.P.); F.Anderson@murdoch.edu.au (F.A.); D.Pethick@murdoch.edu.au (D.W.P.); G.Gardner@murdoch.edu.au (G.E.G.); 2 Australian Cooperative Research Centre for Sheep Industry Innovation, Armidale, New South Wales 2351, Australia; 3 Department of Primary Industries and Regional Development, Perth 6151, Australia

**Keywords:** lamb age, eating quality, consumer, sensory evaluation, sheepmeat

## Abstract

There is an industry wide perception that new season lamb has better eating quality than old season lamb. This study aims to identify differences in consumer eating quality scores between two age classes in lamb. Consumer eating quality scores from eight cuts across the carcass were evaluated from new season (NS; *n* = 120; average age = 240 days) and old season lambs (OS; *n* = 121; average age = 328 days), sourced from four different flocks. Cuts were grilled (loin, topside, outside, knuckle and rump) or roasted (leg, shoulder, rack) and scored by untrained consumers for tenderness, juiciness, liking of flavour and overall liking. There was no difference in eating quality scores between the two age classes for the loin, leg, shoulder and rack. This was similarly shown in the topside with the exception of juiciness scores where NS lambs were higher than OS lambs. There was also a lack of age difference in the outside with the exception of flock 3 where NS lambs scored higher than OS lambs for all sensory traits. Across all sensory traits, OS lambs received on average 2.8 scores lower for the knuckle and 3.1 scores lower for the rump compared to NS lambs. These results show little difference in eating quality between NS and OS lamb, and highlight the potential to develop high quality OS or “autumn lamb” products, with a similar premium price at retail as NS lambs.

## 1. Introduction

Consumer purchasing behaviour when buying meat is driven by eating quality. Eating quality is heavily influenced by animal age, with increased age resulting in decreased eating quality [1,2]. This is mostly due to increases in collagen concentration and crosslinking that occur as animals mature, which decreases muscle tenderness [2,3,4]. Due to its negative impact on eating quality animal age is factored into price grids and grading systems at slaughter.

Sheep age in Australia is currently identified using dentition, with lambs classified as having no permanent incisors in wear, yearlings or hoggets as having 1–2 erupted permanent incisors in wear, and mutton as having 2 or more erupted permanent incisors [5,6]. Within the lamb classification, there are two sub-categories, new season (NS) lamb (approx. 5–8 months old) and old season (OS) lamb (approx. 10–12 months old). Studies that describe eating quality differences between lamb and mutton show that consumers generally prefer lamb [1]. Thompson et al. [2] reported lamb samples receiving significantly higher consumer scores for tenderness, juiciness, liking of flavour and overall liking compared to mutton samples. Although eating quality differences between lamb and mutton are well described, there is little evidence showing clear eating quality differences within younger animals, particularly across multiple cuts. Pannier et al. [7] compared consumer eating quality scores of the loin and topside cuts between lambs (average age: 335 days) and yearlings (average age: 685 days). There was little to no difference in eating quality scores for the loin between the age groups, however the lambs received higher scores than yearlings for the topside. This study suggests that animal age has a greater impact on eating quality in low quality cuts, for example the topside, than high quality cuts such as the loin. This is possibly attributed to differences in muscle function and characteristics, for example collagen solubility, between low quality and high quality cuts.

The impact of animal age on eating quality of cuts other than the loin and topside has yet to be described, particularly within young animals. Despite this lack of evidence, retail marketing of NS lamb, or “young spring lamb” as a high quality product, has resulted in an industry wide perception that NS lamb has better eating quality than OS lamb. Therefore NS lamb receives a premium price at market compared to OS lamb [8]. It is currently unknown if cuts from OS lamb have the same or worsened quality compared to NS lamb, and therefore if the reduced price at market is justified.

This study aims to further explore the relationship between animal age and eating quality across multiple cuts from NS and OS lambs. It is hypothesised that NS and OS lambs will not differ in their consumer eating quality scores for high quality cuts, however the low quality cuts from OS lambs will receive lower scores than those from NS lambs.

## 2. Materials and Methods

### 2.1. Experimental Design and Slaughter Details

Animals were obtained from four commercial sheep flocks across South Australia and Victoria with each flock described in Table 1. NS and OS lambs were balanced across each flock (total *n* = 241), and lambs from both age classes (NS and OS) within each flock genetically linked by similar sires and dams. Both age groups on each property were kept in the same paddock 3 weeks prior to slaughter. Lambs were weighed prior to being transported to a commercial processing plant, where they were held in lairage overnight and slaughtered the following day. All carcasses were subjected to a medium-voltage electrical stimulation [9] and trimmed according to AUS-MEAT specifications [10]. Carcasses were chilled overnight (3–4 °C) before sampling.

### 2.2. Sample Collection and Measurements

Within 1 h of slaughter, hot carcass weight (HCWT) and GR tissue depth (GR) (measured at the girth rib site, which is 11cm from the midline to the lateral surface of the 12th rib, using a standard GR knife) were measured on all carcasses. Ultimate pH was measured at 24 h post-slaughter on the left portion of the *M. longissimus thoracis* et *lumborum* (LTL) (TPS WP-80 v5.2). From the carcass saddle region the left short loin (AUS-MEAT 4880) [10] up to the 12th rib was removed. Eye muscle area (EMA) (mm^2^) was determined by measuring the width and depth of the exposed LTL surface at the 12th rib using a digital calliper. C-site fat depth (mm) was measured 45 mm from the spine along the LTL at the 12th rib on the exposed surface with a digital calliper. From 176 carcasses, 8 different cuts were removed for eating quality analysis. From the remaining 64 carcasses, 2 cuts were removed and used as links, which served as starter samples during the eating quality sessions to prime the palate. Cuts are further described in Table 2. From the total cuts collected, 813 grill cuts were eaten by 1355 consumers in 23 eating quality sessions, and 528 roast cuts were eaten by 880 consumers in 15 eating quality sessions. All grill cuts were sliced into 5 steaks of 15 mm thickness and trimmed for subcutaneous fat and epimysium (silver skin). The leg and shoulder cuts once removed from the carcass were netted using meat netting. The rack cuts were frenched, and the cap fat was removed. All cuts were vacuum-packed and stored at 2 °C for 5 days of aging before being frozen at −20 °C. Cuts were thawed in a 4 °C fridge 24 h prior to sensory testing.

The remaining loin from each carcass was removed for fresh colour, intramuscular fat (IMF) and shear force (SF) measurements. Fresh eye muscle colour was measured on all carcasses approximately 49 h post-mortem on a section of the LL, which was exposed to air for 30–60 min. Lightness (L *), redness (a *) and yellowness (b *) were measured using a Minolta Chromameter, D65 illuminant with a 2° standard observer and 8 mm aperture, as per Pearce et al. [9]. IMF (expressed as a percentage) measurements were taken from a 40 g LL muscle sample. Samples were ground, freeze-dried in a Coolsafe 95-15 Pro (Scanvac, Lillerød, Denmark) and analysed using near-infrared technology (Technicon InfrAlyser 450 (19 wavelengths)) to estimate chemical fat content, as described by Perry et al. [11]. An additional 65 g of loin muscle was collected for shear force testing. Samples were vacuum-packed, aged for 5 days at 1 °C and then frozen at −20 °C until subsequent testing. Packaged frozen samples were cooked in a water bath at 71 °C for 35 min and then cooled in running water for 30 min after cooking. Six cores (approximately 3–4 cm long, 1 cm^2^) from each loin sample were cut and Warner–Bratzler shear force (WBSF) was measured on each core sample using a Lloyd texture analyser with a Warner–Bratzler shear blade fitted [12]. Laboratory processing of loin samples and measurement of WBSF was performed at the University of New England Meat Science Department (Armidale, New South Wales, Australia).

### 2.3. Sensory Testing

The sensory testing protocol for grilled lamb cuts is outlined in Thompson et al [13]. The grilled cuts were cooked on a Silex grill (S-tronic steaker, Silex, Hamburg, Germany), with the top plate set to a temperature of 185 °C and the bottom plate set to 190 °C. Steaks were removed from the grill with an approximate internal temperature of 65 °C, measured with a heat resistance thermometer, rested for 1.5 min and halved before serving.

Leg and shoulder roast cuts were trimmed into a 15 cm × 15 cm block. Cuts were rolled and secured using butchers string prior to cooking. Roast cuts were cooked in an Electrolux 10 tray dry oven and set to a temperature of 160 °C. To achieve an internal temperature of 65 °C, roasts were removed from the oven at an internal temperature of 60 °C and rested for 10 min. Roasts were then sliced into 4 mm samples. Ten suitable slices from each cut were selected for consumer testing. Any external fat and connective tissue seams were removed and slices were trimmed to approximately 50 mm wide × 50 mm long × 4 mm thick. The 10 consumer samples were placed in steel pans which were maintained at a temperature of 50 °C until serving.

Untrained consumers were recruited by an independent company and screened to include individuals aged between 18 and 70 years old. Consumers scored each sample for tenderness, juiciness, liking of flavour and overall liking on a scale of 0 to 100, where a score of 0 indicates a tough, dry, unliked sample, while a score of 100 indicates a very tender, juicy, liked sample. Overall liking scores were used to determine a classification of high quality or low quality for each cut, based on a cut off of 65, which represents a 4-star Meat Standard Australiarating. Each consumer started with a common starter sample that was removed from the link cuts described in Section 2.2. Consumers then tested six consecutive samples over a period of 1 h allocated using a Latin square design [13]. An individual tasting session consisted of 60 consumers that either participated in a grill session or roast session. Overall, 36 cuts were tested within each tasting session, and each cut was sampled by 10 different consumers so that each cut received 10 consumer responses. The study was conducted in accordance with the Declaration of Helsinki, and the protocol was approved by the Ethics Committee of Murdoch University on 11 October 2018 (2018/129).

### 2.4. Statistical Analysis

Linear mixed effects models in SAS (SAS Version 9.1, SAS Institute, Cary, NC, USA) were used to analyse all consumer scores for overall liking, tenderness, juiciness and liking of flavour. The base model for each sensory trait included fixed effects for cut (loin, topside, outside, rump, knuckle, rack, shoulder, leg), age class (new season, old season) and flock within kill group. Animal identification and consumer identification within each consumer session were included as random terms. In addition to the base model, HCWT and GR tissue depth were analysed individually as covariates. All relevant first-order interactions between fixed effects were tested and non-significant (*p* > 0.05) terms were removed in a stepwise manner. In addition, HCWT, IMF%, SF, GR tissue depth, C-site fat depth, EMA, L *, a * and b * were also analysed as dependant variables to determine the phenotypic variability of each trait between the NS and OS lambs. These models included fixed effects for age class and flock within each kill group. Relevant first-order interactions between fixed effects were included and non-significant (*p* > 0.05) terms were removed in a stepwise manner.

## 3. Results

### 3.1. Effect of Lamb Age on Consumer Sensory Scores

Consumer scores for tenderness, juiciness, liking of flavour and overall liking varied between NS and OS lambs within some cuts and some flocks (Table 3 and Table 4).

Within the grill cuts tested, the loin had no difference in scores between NS and OS lambs for all eating quality traits across all flocks. This was similarly seen in the topside with the exception of juiciness scores in flock 2, where NS lambs scored 4.9 scores higher than OS lambs. Within knuckle, rump and outside cuts, there was at least one flock where NS lambs scored higher than OS lambs (*p* < 0.05, Table 4). Within these cuts, these differences ranged between 7–10 points for tenderness, 5–8 points for juiciness and 5–8 points for overall liking. These differences most often occurred within flock 3. Liking of flavour scores did not differ between NS and OS lambs within any of the grill cuts with the exception of the outside from flock 2 where NS lambs scored 5.7 scores higher than OS lambs.

Within the roast cuts the rack and leg had no difference in scores between NS and OS lambs for all sensory traits across all flocks. There was also no age class effect within the shoulder cut for all sensory traits in flocks 2, 3 and 4. There was an age class effect seen in flock 1, however the effect was opposite to all other cuts with OS lambs scoring 5.3, 4.9, 5.2 and 6.8 sensory scores higher for tenderness, juiciness, liking of flavour and overall liking, respectively compared to the NS lambs (*p* < 0.05, Table 4).

These differences described remained mostly unchanged when carcass measurements were included. When corrected for HCWT the effect of age on juiciness scores within flock 1 for the knuckle and rump disappeared, however the magnitude of difference remained the same. There was no change in differences between sensory scores for NS and OS lambs when corrected for GR fat depth.

### 3.2. Carcass Data and Instrumental Meat Quality Measurement

Significant differences in carcass characteristics were found between NS and OS lambs. OS lambs were 82.5 days older and 1.55 kg heavier, had 1.6 mm more GR tissue depth, 0.57% more IMF%, 0.78 mm^2^ more EMA and 0.3 more redness (a *) in the loin than NS lambs (*p* < 0.05 for all measurements, Table 5). These differences were not consistent across flocks, with only flock 3 consistently aligning with this overall trend. In this case the differences between OS and NS lambs also tended to be greatest, with 5.5 kg heavier carcasses, 3.5 mm more GR tissue depth, 1.4 mm^2^ EMA and 1.2% more IMF% (*p* < 0.05 for all measurements, Table 5). Carcass measurements including C-site fat depth, shear force, lightness, yellowness and ultimate pH did not differ between age classes overall; however, some differences did exist within individual flocks. OS lambs from flock 2 and 3 had significantly more C-site fat depth than NS lambs with differences of 1.2 mm and 0.8 mm, respectively (*p* < 0.05). For shear force, only flock 1 displayed a significant difference between age classes with OS lambs having 0.6 kgF less than NS lambs. OS lambs from flock 2 showed more yellowness in the loin with a b * difference of 1.2, while OS lambs from flock 3 had lower ultimate pH with a difference of 0.1 from NS lambs.

## 4. Discussion

### 4.1. Effect of Lamb Age on Eating Quality

There was no consistent effect of age class on consumer sensory scores across all flocks. In partial support of our hypothesis, there was no age class effect for some high quality cuts (loin, shoulder and rack), yet for the knuckle and rump, NS lambs had higher sensory scores than OS lambs. Contrary to our expectations for the low quality cuts (outside, topside and leg) there was little to no difference in consumer scores between age classes.

Most cuts across all flocks showed no difference in consumer scores between NS and OS lambs. This could be attributed to the small differences in age (approximately 3.5 months). This aligns well with other studies that showed no difference in sensory scores of the loin and topside cuts between animals aged 8.5 and 20 months old [1], or between lambs of two dentition categories: lambs that had milk teeth intact, and lambs that had lost milk teeth or had permanent teeth that just erupted [14]. These results suggest that eating quality is not impacted by animal age within lambs, but rather within more mature animals.

The little to no difference in eating quality could also be due to differing genetics between the age classes within each flock. NS lambs are designed to be fast growing for early turn off and are often sired by terminal rams, and terminal or maternal dams. While OS lambs are slower growing and late maturing, often sired by maternal rams and merino dams [15], as reflected in the lambs sourced for this study. It was shown that terminal sired lambs scored lower for both loin and topside tenderness, juiciness, liking of flavour and overall liking in comparison to maternal sired lambs [16]. In the loin, dam breeds also differed in sensory scores with lambs from merino dams scoring 1.9, 1.2, 1.5 and 1.4 scores higher than maternal merino dams for tenderness, juiciness, liking of flavour and overall liking, respectively [16]. It is possible that OS lambs are genetically superior for eating quality and therefore counteract any differences associated with age.

In contrast to most NS and OS cuts that received similar consumer scores, there was a significant age class effect for the knuckle and rump cuts. This was not expected due to their ranking as high quality cuts based on the consumer sensory scores. There are no previous studies comparing consumer scores across sheep ages for these cuts, however is was assumed that cuts deemed as high quality based on consumer scores would reflect the same results as the loin in other studies, as this is a consistently high quality and high scoring cut. Furthermore, Pannier et al. [7] showed a clear variation in the impact of age between a high quality and low quality cut, further reinforcing the notion that cuts with high scores were affected by animal age. However, these results suggest that the impact of animal age on eating quality cannot simply be explained by a ranking of high or low quality based on consumer scores. Differences between age classes in the knuckle and rump may be due to the muscle characteristics of these cuts, such as collagen solubility, rather than the overall quality.

As animal age increases, collagen solubility and tenderness are shown to decrease [4,17,18]. Decreases in collagen solubility vary between cuts, possibly explaining the decreased eating quality only seen in the knuckle and rump cuts of OS lambs. Cross et al. [19] compared collagen concentrations of topside, outside and knuckle cuts within 6 month old and 12 month old lambs. Soluble collagen was lower in the older animals, decreasing the most in the knuckle (5%), followed by the outside and topside (3.3 and 0.5%). In cattle, Girard et al. [20] also found greater decreases in collagen solubility from 6 to 12 months of age for the rump (18.3%) compared to the outside (3.3%). These studies found that with increased animal age, collagen solubility decreases the most rapidly in the knuckle and rump cuts, which therefore may contribute to rapid decreases in tenderness.

There was no difference in sensory scores between NS and OS lambs for the roast cuts. This is most likely due to the cooking method. Cooking temperature and time has significant effects on the heat solubility of collagen [21]. As temperature and time increases connective tissue becomes softer [22,23]. This is attributed to the conversion of collagen to gelatine at high temperatures [24]. Thus, differences seen in sensory scores between NS and OS lambs in the grilled leg cuts (knuckle and rump), which are attributed to decreases in collagen solubility, are mitigated or slowed by the roasting process.

### 4.2. Carcas Data and Instrumental Meat Quality Measures

NS lambs were lighter and leaner when compared to OS lambs, which is not surprising due to the difference in maturity of the two age classes. Older animals that are more mature are often heavier and fatter than younger animals [25]. OS lambs also had higher IMF% values which is in agreement with other reports of IMF% increasing with increased animal age [1,16,26]. Shear force values did not differ between the age classes, which aligns well with the lack of difference in tenderness scores between NS and OS lambs. These results are also in agreement with previous studies that showed no age class effect on loin or topside shear force in lambs of various ages [1,14,27,28]. It is well described that decreases in tenderness that occur with age are due to increases in collagen crosslinks [4,17,18,29]. More crosslinking increases the heat resistance of collagen during the cooking process [4], which in turn results in tougher meat. The lack of shear force and tenderness difference between NS and OS lambs supports the notion that collagen crosslinking does not seem to occur in sheep until at least 12 months old, and potentially older for some cuts.

OS lambs were redder compared to NS lambs, yet still considered acceptable for consumers (L * values of 34 and a * values higher that 14.5 are considered acceptable to consumers) [30]. This is in agreement with Kim et al. [28] and Pethick et al. [1] who demonstrated redder meat in older animals, suggesting increased oxidative fibres and aerobicity as animals age [31].

## 5. Conclusions

This study has shown little to no differences in eating quality between NS and OS lambs across several major cuts of the carcass. This is in contrast to the current perception that NS lambs, or “young spring lambs”, have better quality than OS, carry over lambs. OS lambs should therefore receive the same premium price as NS lambs at retail. This study highlights the potential to develop high quality OS or “autumn lamb” products, with the greatest opportunity in roast cuts. This study also demonstrates that in lambs, cut type is more significant in the impact of eating quality than animal age. Differences in eating quality was only seen within two particular cuts, knuckle and rump, and is attributed to decreased rates of collagen solubility; therefore future work should involve identifying changes in collagen solubility across cuts.

## Figures and Tables

**Table 1:** Location, number, breed, average age and kill group of new season and old season lambs within each flock.

			New Season			Old Season		
Flock	Location	Kill Group	*n*	Sire Type × Dam Breed	Average Age	*n*	Sire Type × Dam Breed	Average Age
1	Lochabar, South Australia	1	30	White Suffolk × White Suffolk	209 ^a^	30	White Suffolk × Merino	298 ^b^
2	Avenue Range, South Australia	2	30	Poll Dorsett × Border Leicester Merino	252 ^a^	30	Border Leicester × Merino	308 ^b^
3	Struan, South Australia	3	30	Poll Dorsett × Border Leicester Merino	250 ^a^	30	Border Leicester × Merino	350 ^b^
4	Greta, Victoria	3	30	Border Leicester composites	252 ^a^	31	Border Leicester composites	357 ^b^
Total			120			121		

Values within rows followed by different letters are significantly different at *p* < 0.05.

**Table 2:** Number and type of cuts collected and eaten by consumers according to cooking method.

Cut	AUS-MEAT Code	Cooking Method	*n* Tested
Knuckle	5072	Grill	163
Loin	5150	Grill	163
Rump	5074	Grill	163
Outside	5075	Grill	163
Topside	5077	Grill	161
Leg	4830	Roast	176
Shoulder	5050	Roast	176
Rack	4748	Roast	176

**Table 3:** Numerator degrees of freedom and F-values for the effects of the base linear mixed effects models of the tenderness, juiciness, flavour and overall liking scores.

Effect	NDF	Tenderness	Juiciness	Liking of Flavour	Overall Liking
Cut	7	284.6 **	155.6 **	117.0 **	169.8 **
Age class	1	2.1	2.7	1.1	1.1
Flock (Kill group)	2	7.7 **	11.8 **	11.6 **	9.7 **
Cut * Age class	7	2.7 *	4.1 **	2.7 *	ns
Flock (Kill group) * Age class	3	3.0 *	1.5	ns	ns
Cut * Flock (Kill group)	21	6.4 **	8.0 **	3.4 **	5.1 **
Cut * Flock (Kill group) * Age class	24	1.8 *	1.6 *	ns	ns

NDF: numerator degrees of freedom; *: *p* < 0.05: **: *p* < 0.001.

**Table 4:** Sensory properties of cuts from new season (NS) and old season (OS) lambs within each flock (least square means ± S.E).

		Tenderness		Juiciness		Liking of Flavour		Overall Liking	
Cut	Flock	NS	OS	NS	OS	NS	OS	NS	OS
Knuckle	1	72.8 ± 1.8	68.7 ± 1.8	68.9 ± 1.7 ^a^	63.9 ± 1.8 ^b^	69.3 ± 1.6	66.5 ± 1.7	70.6 ± 1.7	67.6 ± 1.8
	2	67.1 ± 1.8	64.1 ± 1.8	67.1 ± 1.7	64.1 ± 1.7	67.8 ± 1.6	64.6 ± 1.6	68.0 ± 1.7	65.8 ± 1.7
	3	72.2 ± 1.8 ^a^	64.0 ± 1.8 ^b^	65.8 ± 1.7	62.0 ± 1.7	67.7 ± 1.7	63.3 ± 1.6	70.0 ± 1.7 ^a^	63.4 ± 1.7 ^b^
	4	65.1 ± 1.8	66.9 ± 1.8	64.5 ± 1.7	68.8 ± 1.7	67.3 ± 1.7	67.1 ± 1.7	67.4 ± 1.7	68.5 ± 1.7
Loin	1	64.2 ± 1.8	68.2 ± 1.8	59.1 ± 1.7	62.2 ± 1.8	63.5 ± 1.6	66.6 ± 1.7	63.4 ± 1.7	66.9 ± 1.7
	2	64.3 ± 1.8	67.6 ± 1.8	62.8 ± 1.7	62.8 ± 1.7	65.4 ± 1.6	65.8 ± 1.6	65.6 ± 1.7	66.5 ± 1.7
	3	62.9 ± 1.8	63.1 ± 1.8	61.2 ± 1.7	57.2 ± 1.8	62.7 ± 1.7	62.8 ± 1.7	63.3 ± 1.7	62.5 ± 1.8
	4	64.8 ± 1.8	60.2 ± 1.8	60.8 ± 1.7	59.8 ± 1.7	64.8 ± 1.7	61.3 ± 1.6	65.2 ± 1.7	62.0 ± 1.7
Rump	1	74.4 ± 1.8	72.6 ± 1.8	74.1 ± 1.7 ^a^	69.3 ± 1.8 ^b^	71.8 ± 1.6	69.1 ± 1.7	74.2 ± 1.7	71.4 ± 1.7
	2	70.2 ± 1.7	67.1 ± 1.8	71.1 ± 1.7 ^a^	64.9 ± 1.7 ^b^	70.3 ± 1.6	66.2 ± 1. 6	70.9 ± 1.7 ^a^	66.2 ± 1.7 ^b^
	3	65.8 ± 1.7 ^a^	58.8 ± 1.8 ^b^	60.2 ± 1.7 ^a^	53.7 ± 1.7 ^b^	61.1 ± 1.7	58.4 ± 1.7	61.8 ± 1.7	58.4 ± 1.7
	4	61.5 ± 1.8	62.8 ± 1.8	63.5 ± 1.7	62.6 ± 1.7	63.7 ± 1.7	63.7 ± 1.7	64.1 ± 1.7	64.4 ± 1.7
Outside	1	57.0 ± 1.7	61.4 ± 1.8	64.3 ± 1.7	65.7 ± 1.8	62.6 ± 1.6	63.2 ± 1.7	61.4 ± 1.7	63.9 ± 1.8
	2	53.4 ± 1.8	51.8 ± 1.8	58.3 ± 1.7	56.2 ± 1.7	59.1 ± 1.7	57.8 ± 1.6	57.9 ± 1.7	56.3 ± 1.7
	3	57.3 ± 1.8 ^a^	47.4 ± 1.8 ^b^	60.5 ± 1.7 ^a^	52.5 ± 1.7 ^b^	59.1 ± 1.7 ^a^	53.6 ± 1.7 ^b^	59.7 ± 1.7 ^a^	51.9 ± 1.7 ^b^
	4	56.1 ± 1.7	53.0 ± 1.8	60.1 ± 1.7	59.3 ± 1.7	61.8 ± 1.6	58.9 ± 1.6	60.8 ± 1.7	59.0 ± 1.7
Topside	1	48.7 ± 1.8	49.9 ± 1.8	55.2 ± 1.7	52.9 ± 1.8	54.4 ± 1.6	56.4 ± 1.7	52.8 ± 1.7	54.9 ± 1.8
	2	46.9 ± 1.8	45.5 ± 1.8	55.8 ± 1.7 ^a^	50.9 ± 1.7 ^b^	55.6 ± 1.7	54.2 ± 1.6	52.8 ± 1.7	51.9 ± 1.7
	3	42.7 ± 1.8	38.2 ± 1.8	43.7 ± 1.7	42.3 ± 1.7	48.9 ± 1.7	47.0 ± 1.7	46.3 ± 1.7	44.0 ± 1.7
	4	43.9 ± 1.8	42.1 ± 1.8	50.4 ± 1.7	50.4 ± 1.7	52.3 ± 1.7	53.3 ± 1.7	50.3 ± 1.7	50.5 ± 1.7
Rack	1	71.4 ± 1.8	73.7 ± 1.8	65.6 ± 1.7	69.4 ± 1.7	67.2 ± 1.6	70.4 ± 1.6	67.8 ± 1.7	71.1 ± 1.7
	2	67.3 ± 1.8	68.6 ± 1.8	60.5 ± 1.7	62.7 ± 1.7	64.1 ± 1.6	66.1 ± 1.6	64.8 ± 1.7	67.1 ± 1.7
	3	69.5 ± 1.7	71.0 ± 1.7	63.3 ± 1.6	65.0 ± 1.6	64.3 ± 1.6	64.8 ± 1.6	66.1 ± 1.6	66.7 ± 1.6
	4	69.5 ± 1.7	67.5 ± 1.7	65.4 ± 1.6	64.0 ± 1.6	67.5 ± 1.6	67.6 ± 1.6	68.6 ± 1.6	67.2 ± 1.6
Leg	1	55.3 ± 1.8	58.0 ± 1.8	51.5 ± 1.7	51.7 ± 1.7	57.1 ± 1.6	59.1 ± 1.6	57.1 ± 1.7	58.1 ± 1.7
	2	50.0 ± 1.7	51.6 ± 1.8	46.9 ± 1.7	48.0 ± 1.7	57.0 ± 1.6	54.1 ± 1.6	54.4 ± 1.7	52.3 ± 1.7
	3	54.7 ± 1.7	51.2 ± 1.7	50.1 ± 1.6	46.9 ± 1.6	54.0 ± 1.6	52.7 ± 1.6	54.9 ± 1.6	52.1 ± 1.6
	4	57.8 ± 1.8	58.7 ± 1.7	53.4 ± 1.6	55.0 ± 1.6	58.1 ± 1.6	60.7 ± 1.6	58.5 ± 1.6	60.5 ± 1.6
Shoulder	1	59.7 ± 1.8 ^a^	65.0 ± 1.8 ^b^	57.2 ± 1.7 ^a^	62.1 ± 1.7 ^b^	59.2 ± 1.6 ^a^	64.4 ± 1.6 ^b^	58.5 ± 1.7 ^a^	65.3 ± 1.7 ^b^
	2	67.2 ± 1.8	66.7 ± 1.8	62.9 ± 1.7	64.8 ± 1.7	62.9 ± 1.6	61.3 ± 1.6	64.9 ± 1.7	63.2 ± 1.7
	3	64.7 ± 1.7	60.5 ± 1.7	58.8 ± 1.6	56.5 ± 1.6	56.2 ± 1.6	56.5 ± 1.6	57.6 ± 1.6	56.7 ± 1.6
	4	64.1 ± 1.7	62.6 ± 1.7	62.2 ± 1.6	61.2 ± 1.6	63.9 ± 1.6	62.2 ± 1.6	63.9 ± 1.6	62.7 ± 1.6

Values within rows for each sensory trait followed by different letters are significantly different at *p* < 0.05.

**Table 5:** Carcass characteristics, colour parameters and instrumental measures of new season and old season lambs from each flock (predicted means ± S.E.), and the differences between new season and old season lambs for these measurements.

	Flock	New Season	Old Season	Difference
Hot carcass weight (kg)	1	22.4 ± 0.5	21.75 ± 0.5	0.6
	2	23.04 ± 0.5	22.84 ± 0.5	0.2
	3	21.43 ± 0.5 ^a^	26.94 ± 0.5 ^b^	−5.5
	4	28.61 ± 0.5 ^a^	30.07 ± 0.5 ^b^	−1.5
Girth Rib site tissue depth (mm)	1	10.02 ± 0.7	11.64 ± 0.8	−1.6
	2	16.03 ± 0.7	17.02 ± 0.7	−1
	3	14.98 ± 0.8 ^a^	18.52 ± 0.8 ^b^	−3.5
	4	20.1 ± 0.7	20.28 ± 0.7	−0.2
C-site fat depth (mm)	1	2.52 ± 0.3	2.09 ± 0.3	0.4
	2	2.87 ± 0.3 ^a^	4.12 ± 0.3 ^b^	−1.2
	3	2.84 ± 0.3 ^a^	3.65 ± 0.3 ^b^	−0.8
	4	5.1 ± 0.3	4.53 ± 0.3	0.6
Eye muscle area (mm^2^)	1	13.93 ± 0.4 ^a^	16.1 ± 0.4 ^b^	−2.2
	2	15.3 ± 0.4 ^a^	13.88 ± 0.4 ^b^	1.4
	3	15.37 ± 0.4 ^a^	16.78 ± 0.4 ^b^	−1.4
	4	17.74 ± 0.4	18.69 ± 0.4	−0.9
Intramuscular fat (%)	1	3.31 ± 0.2 ^a^	4.08 ± 0.2 ^b^	−0.8
	2	4.36 ± 0.2	4.84 ± 0.2	−0.5
	3	4.18 ± 0.2 ^a^	5.42 ± 0.2 ^b^	−1.2
	4	5.22 ± 0.2	5.06 ± 0.2	0.2
Shear force at day 5 (KgF)	1	4.43 ± 0.2 ^a^	3.83 ± 0.2 ^b^	0.6
	2	3.72 ± 0.2	3.51 ± 0.2	0.2
	3	4.15 ± 0.2	3.84 ± 0.2	0.3
	4	3.56 ± 0.2	3.62 ± 0.2	−0.1
Lightness (L*)	1	34.17 ± 0.8	32.66 ± 0.9	1.5
	2	34.83 ± 0.8	33.94 ± 0.8	0.9
	3	35.49 ± 0.8	35.98 ± 0.8	−0.5
	4	33.36 ± 0.8	33.73 ± 0.8	−0.4
Redness (a*)	1	4.82 ± 0.2	4.76 ± 0.2	0.1
	2	5.53 ± 0.2 ^a^	6.3 ± 0.2 ^b^	−0.8
	3	5.48 ± 0.2 ^a^	6.13 ± 0.2 ^b^	−0.7
	4	6.28 ± 0.2	6.06 ± 0.2	0.2
Yellowness (b*)	1	16.73 ± 0.3	16.66 ± 0.3	0.1
	2	16.45 ± 0.3 ^a^	17.64 ± 0.3 ^b^	−1.2
	3	16.19 ± 0.3	16.93 ± 0.3	−0.7
	4	18.25 ± 0.3	17.78 ± 0.3	0.5
Ultimate pH	1	5.61 ± 0.02	5.58 ± 0.02	0.03
	2	5.64 ± 0.02	5.65 ± 0.02	−0.01
	3	5.8 ± 0.02 ^a^	5.73 ± 0.02 ^b^	0.1
	4	5.73 ± 0.02	5.76 ± 0.02	−0.03

Values within rows followed by different letters are significantly different at *p* < 0.05.
